# Individual Strategies of Response Organization in Multitasking Are Stable Even at Risk of High Between-Task Interference

**DOI:** 10.3389/fpsyg.2022.860219

**Published:** 2022-04-06

**Authors:** Roman Reinert, Jovita Brüning

**Affiliations:** Department of Psychology and Ergonomics, Technische Universität Berlin, Berlin, Germany

**Keywords:** multitasking, task interference, individual differences, self-organized, dual-tasking

## Abstract

Recently, reliable interindividual differences were found for the way how individuals process multiple tasks (at a cognitive level) and how they organize their responses (at a response level). Previous studies have shown mixed results with respect to the flexibility of these preferences. On the one hand, individuals tend to adjust their preferred task processing mode to varying degrees of risk of crosstalk between tasks. On the other, response strategies were observed to be highly stable under varying between-resource competition. In the present study, we investigated whether the stability of response strategies also persists with increased risk of crosstalk or whether individuals adjust their choice of response strategy, similar to what has been found at the level of task processing modes. Besides, related differences in multitasking efficiency were assessed. For this purpose, 53 participants performed the *Free Concurrent Dual-Tasking* (FCDT) paradigm, which allows them to control their task scheduling and response organization. The participants completed the FCDT paradigm under two conditions including task pairs characterized by either low or high levels of risk of crosstalk. The free choice of task scheduling resulted in the previously found distinct response patterns, best described as *blocking*, *switching* or *response grouping*. Remarkably, we did not find any notable adjustments of strategies of response organization to the extent of crosstalk. However, we observed suspected performance decrements of a switching strategy in the condition of high risk of crosstalk. The results suggest that individual strategies of response organization are stable habits. Further, they illustrate disadvantages of switching vs. blocking strategies of response organization in case of high task similarity.

## Introduction

Interconnected workplaces and a diversification of responsibilities require us to multitask in many of today’s jobs (Green, 2004; Dabbish et al., 2011). Similar challenges arise in our daily lives when we need to operate increasingly complex technology and a multitude of communication channels, which often have to be attended to at the same time (Ophir et al., 2009; van der Schuur et al., 2015; Parry and Le Roux, 2019). In such situations, we need to divide our attention between several activities instead of focusing on one at a time. Yet, as our cognitive resources are finite, so are our capacities for multitasking. Especially when task-similarity is high, working on different tasks in parallel can cause severe task interference and lead to drastically diminished task performance (see, e.g., Allport et al., 1972; Wickens, 1984, 2002). Do people compensate for between-task interference when engaged in multitasking? And if so, can they prevent a performance drop? To answer these questions, we must understand how task interference influences our individual approaches on multitasking.

### Task Interference and Multitasking Performance

It is common ground in multitasking research that performance is worse when working on multiple tasks than under single-tasks conditions. An observation largely attributed to interference between the tasks (see, e.g., Navon and Miller, 1987; Pashler, 1994a; Lien and Proctor, 2002; Wickens, 2002; Monsell, 2003; Schubert, 2008; Vandierendonck et al., 2010). The extent to which tasks interfere essentially depends on how similar they are (see, e.g., Allport et al., 1972, for an early notion on the topic). Still, a distinction can be made between different aspects of the tasks that constitute the tasks’ similarity. In this vein, two lines of research on dual-task performance have focused on different aspects of task similarity, which is primarily due to their fundamentally different assumptions about the general cognitive architecture.

First, research based on capacity-sharing models that assume the existence of multiple resources take a broader perspective on task similarity (see, e.g., McLeod, 1977; Navon and Gopher, 1979; Wickens, 2002). According to the most prominent model, the multiple resource theory (MRT) by Wickens (2002), the extent of task similarity reflects the degree to which tasks require the same cognitive resources. Wickens assumed that tasks differ in the extent to which they require resources from each of four different resource pools (i.e., stages of processing, processing codes, perceptual modalities, and visual channels). In the context of his theory, the extent of between-task interference depends on the degree to which tasks compete for the same cognitive resources. For example, if two tasks consist of verbal stimuli and require manual responses, their concurrent performance will be impaired.

Second, research on task switching and the central stage of response selection often take a more narrowly defined perspective on the relation between task similarity and task interference (see, e.g., Navon and Miller, 1987, 2002; Pashler, 1994a; Hommel, 1998; Koch and Prinz, 2002; Miller, 2006; Koch, 2009; Koob et al., 2021). Whereas task similarity in the MRT refers to differences in the general task characteristics (e.g., the perceptual modality required), task similarity in the second research line relates to the exact stimulus-response (S-R) mappings (e.g., a visual stimulus being a specific digit). When S-R mappings of tasks overlap at the level of stimulus features and/or response features, the resulting between-task interference can produce strong adverse effects on task coordination and performance (Hommel, 1998; Koch, 2009). Between-task interference may arise from what has been termed *crosstalk in concurrent task performance*: It describes “the unwanted transmission of information (i.e., content, such as stimulus or response codes) from one information processing stream (‘information channel’) to the other stream” (Koch et al., 2018, p. 565). Such between-task crosstalk may arise when participants must react to task-specific target stimuli in one task while ignoring information from a second task. One way to enforce crosstalk in task switching studies is by using *bivalent* vs. *univalent* stimuli: If the task-stimuli of one task are also part of the target-stimuli of the other, interference between tasks increases as stimulus-response mappings can be easily confused (Navon and Miller, 1987). Such *bivalent* stimuli evoke considerably more task interference than stimuli that are unambiguously assigned to only one task (*univalent* stimuli; see e.g., Jersild, 1927; Allport et al., 1994). Thus, even stronger task interference can be observed when dual tasks are based on the same set of stimuli than what would be expected with the highest task similarity according to MRT.

### Individual Preferences for Strategies of Task Organization in Multitasking

The aspect of interindividual differences in multitasking has increasingly received attention in recent years (see, e.g., Laguë-Beauvais et al., 2013; Reissland and Manzey, 2016; Heidemann et al., 2020; Kubik et al., 2020; Mittelstädt et al., 2021). Meanwhile, several studies support the existence and relevance of interindividual differences in strategies with which individuals prefer to cope with multiple tasks (e.g., Damos et al., 1983; Reissland and Manzey, 2016; Brüning and Manzey, 2018; Brüning et al., 2020, 2021). As Brüning et al. (2021) pointed out, preferences for multitasking strategies can be distinguished according to the level of task organization at which they occur. In this vein, at a behavioral level of response organization, individual preferences for blocking vs. interleaving response strategies were observed. At a task processing level, individual preferences for serial vs. parallel task processing modes can be differentiated. As the response strategies and the way to identify them are of particular importance to the present study, we will first have a closer look on them before we turn back to the modes of task processing.

**FIGURE 1:**
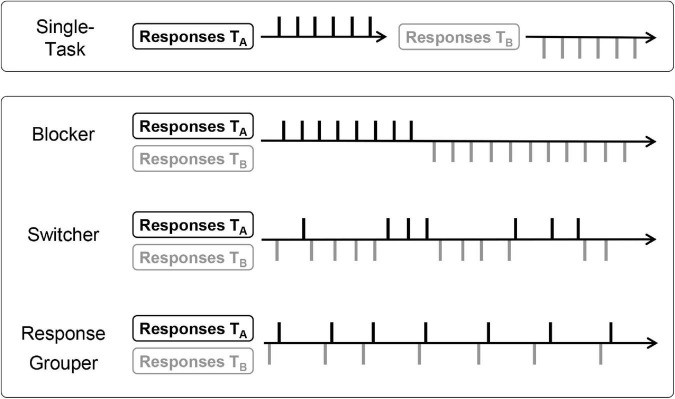
Illustration of characteristic response patterns of each strategy of response organization. Figure taken from Brüning et al. (2020, p. 3). Letters T_A_ and T_B_ refer to Task A and Task B, respectively.

A paradigm designed to identify the individually preferred behavioral strategy is the *free concurrent dual-tasking* (FCDT) paradigm by Reissland and Manzey (2016). A demonstration of the paradigm is provided on the open science framework platform.^1^ The FCDT paradigm enables participants to organize responses according to their natural preferences: Two tasks (A and B) are always visible and there is no fixed order in which they must be solved. Participants therefore can freely choose when to respond to which task, provided they treat both with equal priority. The subsequent identification of the response strategies is based on a detailed analyses of the participant’s individual response patterns (see Brüning et al., 2021). Depending on their proportion of switches and the distribution of inter-response intervals (IRI) in task switches, participants can be classified in three distinct categories: *blocker*, *switcher*, or *response grouper*. Individuals preferring the blocking strategy rarely switch between the tasks (i.e., in less than 10% of trials). By contrast, the switching and response grouping strategies are characterized by frequent task switches, terming them *interleaving strategies*. Specifically, a switching strategy is characterized by frequent but irregular task switches (i.e., about every 4–7 trials). A response grouping strategy corresponds to constant switching between tasks while additionally grouping them into pairs (see “Materials and Methods” section for further details). An illustration of exemplary response patterns corresponding to the different strategies is shown in Figure 1.

Importantly, recent findings by Brüning et al. (2021) indicate that the preferred strategies of response organization and individual preferences for a more serial or parallel mode of task processing are systematically linked. As previous findings on the level of process organization are of relevance for the reasoning in the present study, we will first take a closer look on the individual preferences for processing modes before we turn to known influences of task interference on both levels.

Interindividual differences in the participants’ preference for serial vs. more parallel processing of the very same dual-tasking demands can be assessed using the *task-switching with preview* (TSWP) paradigm (Reissland and Manzey, 2016). A demonstration of the TSWP paradigm is provided on the open science framework platform.^2^ Similar to the alternating-runs task switching paradigm (see, e.g., Rogers and Monsell, 1995), the TSWP paradigm requires participants to work on two discrete tasks A and B in a prescribed alternating order. Critically, while working on three repetitions of the currently relevant task, participants can already see the stimulus of the other task, which must be responded to after the task switch. Thus, participants always have the option to preview the upcoming switch stimulus. The subsequent identification of the preferred processing modes is based on a detailed analyses of the switch and mixing costs in the individual response patterns (see Brüning et al., 2021, for a detailed description). Switch costs reflect prolonged response times in switch trials compared to repetition trials due to time-consuming processes (e.g., reconfiguration of task sets), whereas mixing costs reflect prolonged response times in repetition trials compared to single-task trials. In case participants produce switching costs comparable to those found in classical task switching paradigms without a preview, we can infer that they do not use the preview option. Accordingly, it can be assumed that they process the tasks in a serial manner (i.e., similarly as one would do in classical task switching paradigms). Thus, these individuals are classified as *serial processors*. However, if participants show systematically reduced response times (RTs) in task switch trials without a systematic prolongation in preceding trials (i.e., no mixing costs), we can infer that they use the preview option for overlapping processing. Thus, these individuals are classified as *overlapping processors*. Although overlapping processing entails additional processes (e.g., combatting proactive interference), it allows to compensate for switch costs (Brüning and Manzey, 2018) or can even lead to multitasking benefits (Brüning et al., 2020). Important to the present study, most individuals who applied overlapping processing often preferred switching or response grouping, whereas those who process tasks serially tend to organize their responses in a blocking manner (Brüning et al., 2021).

### Flexibility of Process and Response Organization in Multitasking

Acknowledging that there are individual preferences at both levels of task organization, the question how stable they are, arises. In other words, do humans always persist in the same modes and strategies when they perform multiple tasks irrespective of other factors, or do they adapt their approaches to varying requirements?

So far, the findings resulting from both levels of task organization are rather mixed regarding the flexibility vs. stability issue of individual preferences. At the level of process organization, there is some evidence from research with different paradigms that indicates not only some flexibility but also adaptability to crosstalk.

Such empirical evidence comes, for example, from studies using the psychological refractory period (PRP) paradigm (Lehle and Hübner, 2009; Fischer et al., 2014; Laguë-Beauvais et al., 2015; see Fischer and Plessow, 2015, for a review). The PRP paradigm represents a specific version of a dual-task paradigm comprising two tasks (Task 1 and Task 2), which are presented with varying temporal overlap (stimulus onset asynchrony, SOA). Typically, participants are instructed to respond serially to both tasks, resulting in increased RTs for Task 2 with decreasing SOA (i.e., the so-called PRP effect; Telford, 1931). Moreover, Task 2 can exhibit mutual influence on Task 1, in case both tasks employ overlapping S-R-rules (Hommel, 1998; Janczyk et al., 2018). Importantly, several studies demonstrated that individuals are in fact able to adapt to between-task crosstalk by adjusting the degree of serial processing in a dual-task. For instance, Fischer et al. (2014) found that participants make more use of a serial mode of task processing (i.e., can reduce between-task crosstalk) when tasks appear at a location that is associated with a high compared to low risk of crosstalk. According to Fischer and Plessow (2015), shifting between parallel and serial modes of processing represents a marker of adaptive behavior.

Being flexible in choosing one’s approach on multitasking would come with the advantage of being able to adapt to task characteristics. In this way, performance losses due to high task interference may be prevented. A study addressing this issue relates back to the individual preferences at the process organization level: Brüning and Manzey (2018) demonstrated that the preferred mode of task processing can be adapted to different levels of crosstalk. They had participants perform the TSWP paradigm under two conditions, *high risk of crosstalk* and *low risk of crosstalk*, by using bivalent and univalent stimuli for the tasks, respectively. It was observed that a majority of participants, who were identified as overlapping processors in the condition of low risk of crosstalk changed to a serial mode of task processing in the condition with a high risk of crosstalk (Brüning and Manzey, 2018).

At variance with the outlined observations, other findings from the level of response organization seem to contradict the notion of flexibly adapted preferences in task organization. Brüning et al. (2020) showed that the individual preference for a specific response strategy is not only highly reliable (i.e., consistent within a test session) but also remarkably stable in the face of varying between-task resource competition. They reported that participants persisted in their individual response strategies regardless of the degree to which tasks competed for the same vs. different cognitive processing-code resources. This is especially striking given the apparent link between the two levels of task organization (Brüning et al., 2021) and the flexibility observed at the level of process organization. However, as pointed out above there are notable differences between the task interference induced in the study by Brüning and Manzey (2018) and Brüning et al. (2020). In the former study, task interference arises due to the competition for similar cognitive resources as described in the multiple resource model. By contrast, task interference is way more intense when it is based on crosstalk between tasks with highly similar (S-R) mappings, as in the latter study. Accordingly, one could argue with respect to the findings by Brüning et al. (2020) that, in case of reduced resource competition, an adaptation of response strategies is merely an optional fine tuning for better task efficiency which participants did not deem necessary.

### Current Research

With the present study, we aimed to test whether individual preferences for strategies of response organization remain stable in the face of increased risk of crosstalk. Or in other words: Do people deviate from their preferred behavioral strategies to compensate for between-task interference when engaged in multitasking? To this end, we used the FCDT paradigm and employed two conditions of varying degree of risk of crosstalk with univalent vs. bivalent stimuli, similar to the conditions in Brüning and Manzey (2018). Given the flexibility of processing modes in general and the flexible adjustment of overlapping processing toward more serial processing under increased risk of crosstalk, we expected such flexible adjustment also for response strategies. Especially, as overlapping processing is linked with interleaving strategies, we expected individuals preferring to switch or group their responses under low risk of crosstalk to shift and apply a blocking strategy under high risk of crosstalk to prevent performance decrement. This should be observable in a greater number of participants following a blocking strategy when the risk of crosstalk is high and a negative relationship between risk of crosstalk and switch rate. In addition, we aimed to conceptually replicate a previous finding that a higher switch rate is related to a stronger self-reported preference for multitasking (i.e., a higher polychronicity; cf. Brüning et al., 2020). The individual’s polychronicity reflects the individual’s attitude toward multitasking in real-life situations, which is shaped by past experiences with multitasking scenarios (Slocombe and Bluedorn, 1999; see also Ishizaka et al., 2001). It was, thus, assumed to be predictive for the choice of response strategies in FCDT and was assessed in the present study with the Multitasking Preference Inventory (MPI; Poposki and Oswald, 2010).

## Materials and Methods

### Participants

In total, 57 volunteers were recruited for the experiment. To determine the required sample size, we conducted a-priori power simulations as outlined below. Two datasets were discarded because the participants clearly prioritized one of the tasks (i.e., one task was performed more than 1.5 times more often as the other). One dataset was rejected due to the participant’s high error rate (ER) of above 15%. The dataset of one participant was excluded because the response strategy could not be identified based on the applied classification criteria.^3^ Of the 53 remaining participants (34 females, *M* age = 27 years, *SD* = 4 years, range = 20–35 years), 48 were right-handed, three left-handed and one ambidextrous. All participants had normal or corrected-to-normal vision, spoke German at native language level and their mother tongue was based on the Latin alphabet.

Power calculations for detecting the effect of interest with the McNemar test (i.e., number of participants changing their strategy) were conducted via Monte Carlo simulations prior to data collection. To this end, we defined a contingency table with the probabilities of p(b), p(c), and p(IL). Here, p(b) is the probability that a participant favors a blocking strategy under the condition of low risk of crosstalk and an interleaving strategy when risk of crosstalk is high. Conversely, p(c) is the probability that a participant favors an interleaving strategy in the condition of low risk of crosstalk and a blocking strategy under high risk of crosstalk. Finally, p(IL) is the marginal probability that a participant favors an interleaving strategy under low risk of crosstalk conditions, which was set to 50% (inspired by previous results; Brüning et al., 2021). In the simulation, we varied *p*(*b*) ∈ {0, 0.05, 0.1}, *p*(*c*) ∈ {0, 0.025, 0.05,…,0.5}, and the sample size. To determine the power of one combination of these parameters, we simulated 10,000 McNemar tests by randomly drawing samples based on the defined probabilities. Even after excluding four participants from our overall sample size of 57 participants, the McNemar tests were significant in 80% of the time, for *p*(*c*)≥0.35 and *p*(*b*) = 0.1, *p*(*c*)≥0.25 and *p*(*b*) = 0.05, *p*(*c*)≤0.125 and *p*(*b*) = 0. Note that in Brüning and Manzey (2018) $\hat{p\,\left(c\right)}$ and $\hat{p\,\left(b\right)}$ were found to be 0.38 and 0. Thus, our sample size of 53 participants should be sufficient to detect the effect of interest.

Furthermore, the experiment adhered to the standards set by the local ethics committee. Informed consent was obtained from all individual participants included in the study. Participants received either 15 € or course credit as compensation. Additionally, a performance-based incentive of up to five Euro was paid to motivate participants to work both quickly and accurately.

### Tasks

**FIGURE 2:**
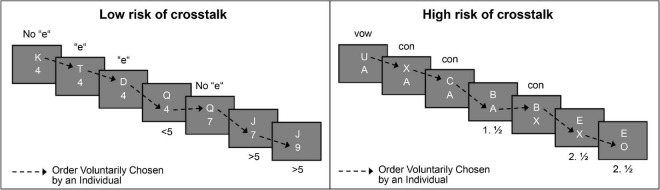
Schematic of the free concurrent dual-tasking (FCDT) paradigm. The figure shows an exemplary trial sequence of a voluntarily chosen task order. The left panel shows the condition of low risk of crosstalk with the corresponding letter (spoken “e” vs. no “e”) and digit task (</>5). The right panel depicts the condition of high risk of crosstalk with the corresponding two letter tasks (vowel vs. consonants; first vs. second half of alphabet).

Four simple choice-reaction tasks were used in the FCDT paradigm. Participants either worked on the tasks separately during single-task blocks or concurrently during dual-task blocks. In dual-task blocks, the tasks were combined to two pairs for all participants such that they reflect conditions of low and high risk of crosstalk, as depicted in Figure 2. The first pair of tasks consisted of a digit classification task and a letter classification task (i.e., univalent stimuli), therefore involving low risk of crosstalk during concurrent performance. The digit classification task required participants to decide if the presented digit is less (1, 2, 3, 4) or greater than five (6, 7, 8, 9). In the letter classification task, the displayed consonant had to be categorized as to whether their pronunciation includes an ‘‘e’’ (letters D, P, T, and W), or not (letters H, K, J, and Q).^4^ The second task pair consisted of two letter classification tasks (i.e., bivalent stimuli), which were based on the same set of letters (A, B, C, E, O, U, X, Z), resulting in high risk of crosstalk. When working on the “alphabet-task,” participants had to indicate whether the presented letter appears in the first (A, B, C, E) or the second half of the alphabet (O, U, X, Z). In the “vowel-consonant-task,” the letters had to be identified as vowels (A, E, O, U) or consonants (B, C, X, Z). Participants received on-screen instructions for the tasks to read them self-paced. They were instructed to produce as many correct responses as possible in the given time in order to optimize performance and thus maximize the performance-based bonus payment. They were also instructed to perform both tasks with the same priority in dual-task blocks.

### Stimuli and Apparatus

During the FCDT, all digit and letter stimuli were displayed in white (RGB 255, 255, 255; font size = 24 pt) and centered against a gray background (RGB 128, 128, 128) on an Asus PB248Q LCD screen (1,280 × 1,024 px, sampling with 60 Hz). In dual-task blocks, the stimuli were presented in vertical arrangement with close spatial proximity (distance = 16 pixels), enabling concurrent perception of the two stimuli without eye movements. Participants responded by pressing assigned keys on a standard keyboard, by using their respective index and middle fingers. Each task was assigned to one hand. For the left hand, participants pressed the keys “A” and “S.” For the right hand, they used the keys “K” and “L.” Task-hand assignment was counterbalanced between participants. The keys were covered with colored stickers to facilitate recognition. A custom-made JAVA software managed stimulus presentation and timing. Stimuli were drawn randomly such that they did not appear twice in direct succession and that an equal distribution of both possible responses was guaranteed. All stimuli were shown until a response was recorded. Upon response, the displayed task stimulus was immediately replaced by the next (response-stimulus interval = 0 ms), while the stimulus of the other task remained (see Figure 2).

### Additional Measure: Polychronicity

The degree to which an individual is polychronic was assessed with the Multitasking Preference Inventory (MPI; Poposki and Oswald, 2010). The MPI contains statements that describe a person’s personal preferences for performing multiple tasks in their daily life (e.g., “I prefer to work on several projects in a day, rather than completing one project and then switching to another”). It comprised 14 items based on a 5-point Likert scale with a higher sum score reflecting a higher degree of polychronicity. The entire scale had a Cronbach’s alpha reliability coefficient of 0.87. To examine the relationship between polychronicity and the response strategies, simple regression analyses were run for each crosstalk condition to test if MPI scores predicted mean switch rates.

### Design

All participants were tested in the conditions of high and low risk of crosstalk. Based on their performance in the FCDT paradigm, participants were categorized regarding their strategy of response organization into groups of blocker, switcher, or response grouper, separately for each condition. Thus, the design of the study corresponded to a 2 × 3 mixed factorial design, comprising the within-participants factor risk of crosstalk (low vs. high) and the between-participants factor response strategy (blocker vs. switcher vs. response grouper).

### Procedure

Participants completed an online version of the MPI 2 days before performing the FCDT paradigm in the laboratory at the Technische Universität Berlin. In the laboratory, at most two participants were tested simultaneously at individual PC workstations. These were separated by opaque screens and participants were provided with earplugs to minimize distractions. All tasks were performed in blocks for a fixed time (e.g., 60 s), that is, tasks could not be completed in a shorter timeframe. Note that this procedure allows to calculate the performance efficiency not only in terms of costs and benefits for each trial but as a general task throughput (i.e., by comparing the number of dual- and single task trials).

The experiment was divided into two parts, representing the conditions high and low risk of crosstalk, the order of which was counterbalanced. In both parts, preceding the experimental runs, participants were familiarized with the single-tasks for 30 s and practiced the single-tasks for another 60 s to account for initial practice effects. The order in which the single tasks were introduced was balanced across participants. Then, the FCDT procedure for dual-task blocks was introduced similarly but with longer periods for familiarization (60 s) and training (120 s). Subsequently, each part of the experiment included three experimental runs, which consisted of two 120 s dual-task blocks and two 60 s single-task blocks. While dual-task blocks always preceded the single-task blocks, the order in which the single-task blocks were tested was alternated. After every block, participants received feedback on their performance for 5 s, including the number of correct and wrong responses and the percentage of correctly solved trials. Upon completion of a block, the next block started automatically. There were mandatory breaks of 60 s between runs, as well as a break of at least 120 s after the first half of the experiment. In total, the experiment lasted about 90 min.

### Data Analysis

#### Data Structure and Preprocessing

Three different trial types were considered when analyzing the data: single-task trials, repetition trials, and switch trials. Repetition trials were defined as those trials in which participants performed the same task as on the trial before, whereas a switch trial was present if the current trial involved a different task than the trial before. Repetition and switch trials in the dual-task blocks were classified *post-hoc*, as the participants had the freedom to repeat or switch the tasks. Participant’s inter-response intervals (IRIs),^5^ defined as the time interval between two subsequent responses, were calculated for each correct response. Besides, ERs were computed as the number of incorrect responses divided by the total number of responses per block, task, and participant.

The subsequent exclusion of outliers was performed in two steps. First, all trials with an IRI longer than 5 s were excluded. Second, we discarded trials slower than two *SD*s^6^ from the mean IRI of the respective task and trial type per block within each crosstalk condition. As a result, 4.7% of trials were excluded per participant on average (*Min* = 3.2%, *Max* = 6.8%, *SD* = 0.8%). In the fixed time frames, participants performed on average 517 single-task trials (*Min* = 350, *Max* = 645, *SD* = 69.6) and 949 dual-task trials (*Min* = 669, *Max* = 1,247, *SD* = 158) in the condition of low risk of crosstalk. In the condition of high risk of crosstalk, 490 single-task trials (*Min* = 280, *Max* = 614, *SD* = 73.3) and 853 dual-task trials (*Min* = 483, *Max* = 1,210, *SD* = 200) were performed on average.

#### Identification of Individually Preferred Strategies of Response Organization

Following the criteria devised by Brüning et al. (2021), response strategies were identified by means of a *post-hoc* analysis of switch rates and IRI distributions for both crosstalk conditions, separately. Switch rates were calculated as the number of switch trials in relation to the maximum number of possible switches that could have been made given the total number of completed trials in both tasks. Participants who exhibited a switch rate of less than 10% were classified as blocker. This criterion was previously derived to describe the observation that some individuals tend to work on the same task for a longer period, before switching to the other task (i.e., >9 repetitions, on average). It indicates that such individuals minimize the number of task switches but does not exclude that they, although rarely, commit some. In this sense, the criterion was chosen to be necessarily low (i.e., reflecting that task switches are a rare event) and at the same time sufficiently high (i.e., to allow for few switches). In contrast, participants producing a switch rate greater than 10% combined with an unimodal distribution of IRIs in switch trials were classified as switcher. If participants not only switched more than they repeated the tasks (i.e., switch rate > 50%), but IRIs in switch trials were also bimodally distributed, they were classified as response grouper. This bimodal distribution results from prolonged switch IRIs followed by very short IRIs, reflecting that response grouper first process both stimuli internally and then respond to the tasks in short succession. Hartigan’s dip test (Hartigan and Hartigan, 1985) was used to test if IRIs in switch trials deviated from an unimodal distribution or not. To compensate for the test’s high sensitivity, the assumption of unimodality was only rejected if a *p*-value lower than 0.001 was obtained. Additionally, the data was visually inspected to verify bimodal distributions for all cases for which the dip test became significant.

#### Analysis of Multitasking Efficiency

Each participant’s multitasking efficiency was assessed per condition of crosstalk by means of the overall dual-tasking performance efficiency (ODTPE) measure as described by Brüning et al. (2020):


$$O\,D\,T\,P\,E=100\times \left[\left(\frac{\left(n\,C_{A\,\_\,d\,u\,a\,l}\right)}{\left(n\,C_{A\,\_\,s\,i\,n\,g\,l\,e}\right)}+\frac{\left(n\,C_{B\,\_\,d\,u\,a\,l}\right)}{\left(n\,C_{B\,\_\,s\,i\,n\,g\,l\,e}\right)}\right)/2\right]-100$$

The ODTPE relates the number of correct trials in dual-task blocks to the number of correct responses in the subsequent single-task blocks. Resulting ODTPE scores can be interpreted as percentages of performance change, with negative values indicating a performance loss and positive values pointing to a performance gain in dual- compared to single-task blocks (see Appendix for a detailed description). Thus, the ODTPE represents a straightforward throughput measure.

## Results

### Shifts in Individually Preferred Strategies of Response Organization

In both crosstalk conditions, the three response strategies, blocking, switching and response grouping could be observed, although to varying proportions. In the condition of low risk of crosstalk, 31 participants were identified as blocker, 19 chose a switching approach, and three grouped their responses. When working on tasks with high risk of crosstalk, 32 participants followed a blocking strategy, 18 were classified as switcher, and again three grouped their responses. Inspecting the whole sample, remarkable 91% of the individuals chose the same response strategy irrespective of the degree of between-task crosstalk. The few transitions that took place were in opposed directions (see Table 1). Of the five individuals changing their strategy, three shifted between switching in the condition of low risk of crosstalk and blocking when the risk of crosstalk was high. The other two participants shifted between blocking in the condition of low risk of crosstalk and switching when the risk of crosstalk was high. As can be expected, the high stability of the choice of response strategies was reflected in a non-significant exact Bowker test, χ^2^ (*n* = 5) = 0.2, *p* = 1.

**TABLE 1:** Contingency table of applied strategies of response organization in both crosstalk conditions.

		High risk of crosstalk			Total
	Strategy	Blocker	Switcher	Response grouper	
Low risk of crosstalk	Blocker	29	2	0	31
	Switcher	3	16	0	19
	Response grouper	0	0	3	3
Total		32	18	3	53

**FIGURE 3:**
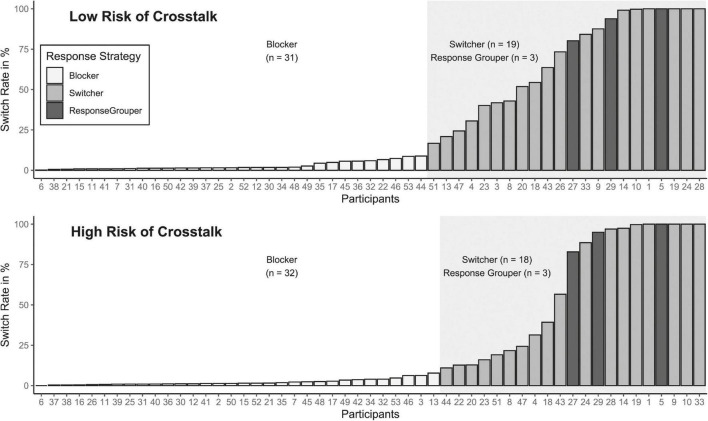
Individual switch rates in percentage and identified strategies for both crosstalk conditions. Switch rates indicate the number of performed switches in relation to the total number of possible switches and are displayed in ranked order.

Because the categorization approach may have masked more subtle effects of increased risk of crosstalk on the switch rate, we next compared the switch rate between crosstalk conditions independent of response strategies. Thus, the according paired *t*-test was based on the 48 participants who consistently used either of the response strategies in both crosstalk conditions. The paired *t*-test was non-significant, *t*(47) = −1.79, *p* = 0.080, $\hat{d}$ = −0.26, indicating no differences in the switch rates of individuals with stable preferences between the crosstalk conditions.^7^ Having obtained a null-effect with frequentist inferential statistics, we also calculated Bayes factors for this within-participants comparison, which led to anecdotal evidence for the null, *BF*_01_=1.47. Figure 3 shows an overview of the individual’s switch rates and associated response strategies in each crosstalk condition. Descriptively, a slight difference between the switch rates of the switcher group in the condition of low compared to high crosstalk becomes apparent.

The means and standard errors (*SE*) of switch rates per response strategy are listed in Table 2. In accordance with the classification procedure, mean switch rates clearly differed between strategies across conditions and displayed a characteristic ranking: Blocker exhibited by far the lowest mean switch rates, switcher performed more than half of all possible switches on average, and response grouper achieved mean switch rates close to 100%.^8^ To further inspect whether the degree of crosstalk had a more subtle effect on the switch rates within the groups of preferred response strategies, we exploratively compared the switch rates within groups across conditions. Due to the small number of response grouper and the fact that this strategy is foremost characterized by the bivalent distribution of IRIs in switch trials, we do not consider them for this analysis. The *t*-tests for switcher and blocker are Bonferroni-Holm corrected. Switcher showed the biggest reduction in mean switch rate ($\hat{\Delta }$ = 5.7%), but this was accompanied by the largest variation in the data (*SE*_*low*_ = 7.8%, *SE*_*high*_ = 9.4%). Neither switcher, *t*(15) = −1.65, *p* = 0.119, $\hat{d}$ = −0.41, nor blocker, *t*(28) = −1.83, *p* = 0.077, $\hat{d}$ = −0.34, showed a significant reduction of their switch rates in the condition of high compared to the condition of low risk of crosstalk. A complementary analysis using Bayes factors led to anecdotal evidence for the null in both cases (Switcher: *BF*_01_=1.28; Blocker: *BF*_01_=1.16). Thus, a higher risk of crosstalk did not affect the degree of switching in the strategies of response organization in any statistically significant way.

**TABLE 2:** Comparison of mean switch rates between crosstalk conditions as a function of response strategy.

	Switch rate in %			
	Low risk of crosstalk		High risk of crosstalk	
Strategy	*M*	*SE*	*M*	*SE*
Blocker	2.5	0.4	1.9	0.3
Switcher	68.4	7.8	62.7	9.4
Response grouper	91.3	5.9	92.6	5.1

### Multitasking Efficiency

**FIGURE 4:**
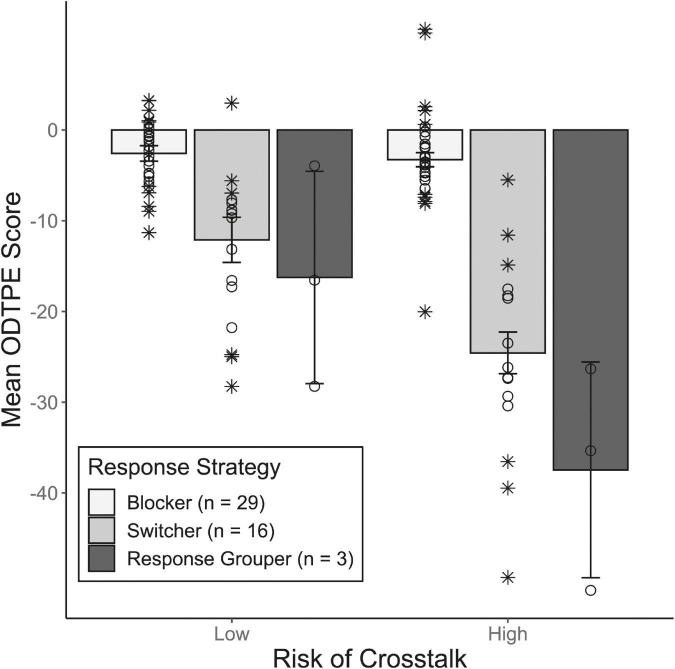
Mean overall dual-task performance efficiency (ODTPE) scores of each response-strategy group in both crosstalk conditions (calculated on trimmed means). Error bars indicate ± one standard error calculated on trimmed means, separately for each Group × Crosstalk Condition. Circles/crosses represent individual data included/excluded in the calculation of trimmed means (20% criterion).

To investigate if increased risk of crosstalk negatively impacted on dual-task performance, differences in the groups’ mean performance efficiency scores (i.e., ODTPE, see Appendix) were compared between crosstalk conditions. Figure 4 illustrates the mean ODTPE scores grouped by response strategy for both conditions. The exact *M*_*t*_ and respective standard errors (*SE_*t*_)* of the ODTPE scores is presented per response strategy and condition of crosstalk in Table 3. As mentioned above, the group of response grouper was too small to be compared with the other groups and will be inspected separately. The resulting groups of blocker and switcher although being considerably larger, differed notably in size. Therefore, a robust implementation of a heteroscedastic mixed ANOVA based on trimmed means (*M*_*t*_) suggested by Mair and Wilcox (2020) was used to compare performance efficiency scores across crosstalk conditions, using the R package WRS2. Besides, the variance between both groups differed markedly with a ratio of variance of about 5. To counteract this issue, we will apply a more conservative alpha level of 0.005, following suggestions by Blanca et al. (2018). The ANOVA revealed a significant main effect of groups, *F*_(1, 21.32)_ = 54.35, *p* < 0.001, a significant main effect of crosstalk, *F*_(1, 22.7)_ = 24.89, *p*< 0.001, as well as a significant Group × Crosstalk Condition interaction, *F*_(1, 22.7)_ = 19.91, *p*< 0.001. According to the robust measure of effect size $\hat{\xi }$ (see *yuen.effect.ci* function of WRS2), the difference between blocker and switcher groups could be considered large ($\hat{\xi }$ = 0.83). To further validate the between-participants effect, we additionally performed a Welch *t*-test. In particular, we first contrasted ODTPE scores between conditions of low and high risk of crosstalk, and then compared these differences between switcher and blocker. The corresponding *t*-test turned out to be significant as well, *t*(20.1) = 4.41, *p* < 0.001, $\hat{d}$ = 0.47.

**TABLE 3:** Comparison of mean overall dual-task performance efficiency (ODTPE) scores between crosstalk conditions.

	ODTPE							
	Low risk of crosstalk				High risk of crosstalk			
Strategy	*M*_*a*_	*SE*_*a*_	*M*_*t*_	*SE*_*t*_	*M*_*a*_	*SE*_*a*_	*M*_*t*_	*SE*_*t*_
Blocker	−2.8	0.7	−2.6	0.9	−3.0	1.1	−3.3	0.8
Switcher	−13.0	2.1	−12.1	2.5	−25.2	2.7	−24.6	2.3
Response grouper	−16.2	7.0	−16.2	11.7	−37.5	7.1	−37.5	11.9

*Note that standard errors (SE_a_, SE_t_) are provided based on 20% trimmed means (M_t_) that were used for the robust ANOVA and on arithmetic means (M_a_) for comparison.*

As becomes evident from Table 3 and Figure 4, blocker stood out as they achieved the best results across conditions. Their mean ODTPE scores were only slightly negative in both conditions, meaning that they accomplished almost the same throughput as if they had worked on both tasks under single-task conditions. In comparison, switcher exhibited moderate performance losses in the condition of low risk of crosstalk but extensive losses when risk of crosstalk was high, resulting in a pronounced performance gap between blocker and switcher in the latter condition ($\hat{\Delta }$ = 21.3).

The group of response grouper showed moderate performance losses comparable to those of switcher in the condition of low risk of crosstalk (*M*_*t*_ = −16.2). In the condition of high risk of crosstalk, the performance gap between response grouper and blocker was even more pronounced than that observed between switcher and blocker (response grouper vs. blocker: $\hat{\Delta }$ = 34.2). However, given the small size of this group (*n* = 3), we refrained from testing any differences with the other two groups.

### Influence of Polychronicity on the Tendency to Switch Between Tasks

The regression analyses testing the influence of polychronicity on mean switch rates included data records of all participants except for one, who had to be excluded due to missing data. A positive linear relationship of MPI score and mean switch rate could not be confirmed, under neither crosstalk condition [low: *t*(50) = −0.12, *p* = 0.902, $\hat{R^{2}}$< 0.01; high: *t*(50) = 0.17, *p* = 0.867, $\hat{R^{2}}$< 0.01]. Additionally, there was no interaction of MPI score and risk of crosstalk [*t*(50) = −0.83, *p* = 0.41, $\hat{R^{2}}$ = 0.01).^9^ Interested readers can find a plot of the raw data including the regression lines in Supplementary Figure 1.

## Discussion

The aim of the present study was to investigate whether individually preferred strategies of response organization are flexibly adapted to different degrees of crosstalk, and how efficient each strategy can be applied. For this purpose, participants were tested in the FCDT paradigm with univalent task stimuli in a condition of low risk of crosstalk and with bivalent task stimuli in a condition of high risk of crosstalk condition. Except for one person, the participant’s chosen strategies of response organization could be unambiguously classified in accordance with the criteria presented in Brüning et al. (2021). As expected, all three approaches to response scheduling, that is blocking, switching and response grouping were found. This widely replicates findings of previous studies reliably discriminating three approaches to response organization in multitasking situations (Damos and Wickens, 1980; Damos et al., 1983; Reissland and Manzey, 2016; Brüning et al., 2020).^10^

### Flexibility of Individual Strategies of Response Organization

Across both crosstalk conditions, most participants seemed to prefer a blocking strategy (*n*_*low*_ = 30, *n*_*high*_ = 32), while fewer acted as switcher (*n*_*low*_ = 19, *n*_*high*_ = 17), and a negligible number grouped their responses (*n*_*low*_ = 3, *n*_*high*_ = 3). As was expected, almost all individuals who preferred a blocking strategy under low risk of crosstalk showed the same preference under high risk of crosstalk. Yet unexpectedly, also participants who preferred an interleaving approach in the condition of low risk of crosstalk did not change to a blocking strategy under high risk of crosstalk. Similarly, an analysis of the underlying continuous parameter, that is, the individual’s mean switch rates also revealed no significant effect of crosstalk and at least anecdotal evidence for the null.

The observed lack of adaptation is insofar surprising as it conflicts with previous research suggesting that humans are in principle able to adapt their way of processing to different degrees of interference in multitasking situations. Findings that point to a flexibility of processing modes and an adaptability to specific task requirements were reported by various studies (e.g., Luria and Meiran, 2005; Lehle and Hübner, 2009; Fischer et al., 2014; Janczyk, 2016; Brüning and Manzey, 2018; see Fischer and Plessow, 2015, for a review). For example, Brüning and Manzey (2018) found an effect of risk of crosstalk on applied processing strategies in the TSWP paradigm, using exactly the same tasks and stimuli as we did in the present study. They observed a pronounced shift from overlapping processing to serial processing with increased risk of crosstalk. Moreover, the systematic link found between a preferred overlapping processing mode and an interleaving response strategy, would have led us to expect a corresponding adaptation at the behavioral level. Furthermore, there are studies showing that individuals possess some adaptability in their task selection behavior (e.g., Nijboer et al., 2013; Mittelstädt et al., 2019; Monno et al., 2021; Spitzer et al., 2021). In particular, Nijboer et al. (2013) had participants perform a primary task (multicolumn subtraction) concurrently with a secondary task which they could pick freely from trial to trial (tone counting vs. tracking task). They found that participants would choose the task that minimized interference in order to optimize performance. Although the participants were not allowed to choose when to work on the primary or the secondary task (i.e., to self-organize their responses), it allows for the following conclusions: People are aware of between-task crosstalk and its implications for multitasking performance and can, in principle, make the right choices to reduce interference between tasks. A finding that is also consistent with research from introspection in multitasking showing that people are aware of their switch costs (Bratzke and Bryce, 2019). Besides, it was shown that individuals are even able to take these costs into account in their task selection behavior (Mittelstädt et al., 2018, 2019, 2021; Monno et al., 2021).

Why, then, did participants not adapt their strategies of response organization to the degree of crosstalk in this study? One could argue that the task pairs did not differ enough in their degree of interference. Yet, this appears implausible considering that Brüning and Manzey (2018) observed an effect using the very same tasks. Thus, it seems reasonable to assume that the same tasks should have led the participants to adapt their strategies of response organization, as well. Interestingly, Brüning et al. (2021) also noted that the correlation between preferred processing modes and strategies of response organization was not perfect. They assumed that both reflect aspects of multitasking that could, to some degree, vary independently of each other. Similarly, Pashler (1994b) showed using the PRP paradigm that serial processing at the stage of response selection can still be associated to interleaving strategies of response organization (i.e., response grouping).

However, an imperfect fit between the individuals’ processing mode and preferred response strategy might only partly explain why participants did not show the expected adaptations in this study. More likely the observation of a lacking adaptability of the response strategies seems to reflect a characteristic of these preferences at the behavioral level of task organization in multitasking. In this sense, the obtained results are well in line with more recent findings reported by Brüning et al. (2020). The authors found a high stability of the preferences for response strategies when the tasks differed considerably in their competition for the same cognitive resources (cf., Wickens, 2002). Only some transitions occurred between interleaving strategies when effector-related resources were separated, whereas the large majority of participants persisted in their way of response scheduling. Notably, an adaptation of response strategies in the setting by Brüning et al. (2020) would merely have been an optional fine tuning for better task efficiency. In contrast, an adaptation of the response strategies in the present study would have been necessary to avoid a high loss in performance efficiency. In this vein, the observed stability of the preferences for response strategies even under intense dual-task interference further underlines the notion that they might represent a rather habitual behavior which seems not to be changed easily. It might be an interesting endeavor to test in future studies whether the applied response strategies can be changed by explicit instruction or reward. However, if according potential changes are only short-term in nature (i.e., individuals revert back to their previous behavior), it would rather strengthen the view that the response strategies resemble habitual behavior (Mazar and Wood, 2018, p. 16).

Lastly, one might speculate how the response strategies align with the two styles of metacognitive control suggested by Hommel (2015). Recently, Brüning et al. (2020) suggested that the preference for blocking might directly reflect a manifestation of a persistent control state, whereas the preference for interleaving might reflect a flexible control state. That is, an individual who uses a blocking strategy seems to prefer an approach that concentrates on the relevant information of the current task and suppresses irrelevant information. In contrast, an individual who uses an interleaving strategy seems to prefer an approach that focuses “on flexibility and facilitates switching between alternative possibilities and actions but increases the possibility of distraction and dysfunctional cross talk between cognitive representations.” (Hommel, 2015, p. 44). In this vein, it further strengthens the notion that people “do not choose control policies randomly or only according to external demands” but rather possess strong “default values that can be biased toward the persistence or the flexibility pole” (Hommel, 2015, p. 49). In addition, the present study further supports the idea that “people can acquire particular personal metacontrol styles that predict important characteristics of their performance” (Hommel, 2015, p. 50).

### Multitasking Efficiency

The negative impact of increased task interference on multitasking performance independent of the response strategy is in line with findings of numerous studies in multitasking research (e.g., Allport et al., 1994; Rogers and Monsell, 1995; Koch, 2008; Lehle and Hübner, 2009; see Kiesel et al., 2010, for a review). This result confirms that the conditions of low and high risk of crosstalk were successfully implemented.

More importantly, the multitasking efficiency achieved with the response strategies was differentially affected by the task interference. The virtually unaffected performance of blocker can be attributed to their strategy of minimizing the number of switches between tasks. With few task switches only few task-set reconfigurations are necessary. Moreover, this strategy supports task shielding and thus reduces the risk of overlap between tasks. Thereby, it poses minimal demands for executive control so only the requirement of maintaining two different task sets may cause interference, eventually leading to the low costs observed (see, e.g., Allport et al., 1994; Koch et al., 2005; Rubin and Meiran, 2005; Poljac et al., 2009).

In contrast, participants who organized their responses in an interleaving manner performed worse than blocker even under low task interference. This lower performance is likely due to the higher requirements that an interleaving strategy poses for executive control. Frequent task switches render to monitor and evaluate one’s own performance more effortful as is supported by various findings (e.g., Arrington and Logan, 2004, 2005; Kübler et al., 2018; Rieger et al., 2021). Apparently these demands are even stronger than the potential benefits of overlapping processing, which was observed to co-occur with interleaving strategies under low task interference (cf., Brüning et al., 2021). With the higher risk to confuse stimulus-response mappings (see, e.g., Navon and Miller, 1987) the performance of interleavers became even worse under high task interference. This advanced performance drop can likely be attributed to the increased requirement to combat proactive interference.

### Lack of Relation to Polychronicity

The connection between an individuals’ self-reported polychronicity and their preference for a behavioral response strategy could not be conceptually replicated. This result was somewhat puzzling as it contradicts previous findings by Brüning et al. (2020), which indicated a positive correlation independent of the degree of resource competition. However, this does not necessarily challenge the predictive value of polychronicity for response strategies in task organization *per se*. As the authors of the MPI pointed out, polychronicity describes a stable tendency to “… perceive multitasking as enjoyable and rewarding rather than stressful,…” (Poposki and Oswald, 2010, p. 247). In this context, König et al. (2005) emphasized that polychronic individuals might be less concerned with performance consequences, but “… simply like the constant changes of work involved in multitasking.” (p. 261). The latter addresses a decisive difference between the multitasking setup used by Brüning et al. (2020) and the one used in the present study. The former involved tasks with a higher variety in task rules, more visually enriched stimuli and different responses. In contrast, the tasks in the present experiment involved more similar task rules and highly similar stimuli and responses, which consequently might not have induced enough variance to reveal this relationship. As the present study did not find a connection between individual polychronicity and actually applied strategies, further research should be undertaken to investigate the importance of task characteristics in this relation.

## Conclusion and Outlook

The results of the present study strengthen the view that individual preferences for response strategies are highly stable, independent of the degree of task interference. This general stability is consistent with previous observations that participants choose their response strategy in the first 2 min of practice (Damos and Wickens, 1980).

However, the remarkable stability contrasts clearly with the adaptations observed for processing modes. The exact cause of the difference in stability between the two levels of task coordination cannot yet be clarified based on the data obtained. A possible source for this difference that should be considered in future research, might be that the two levels of task coordination are differentially influenced by other individual factors. First, the flexibility at the task processing level might be fueled by differences in executive functions of the individuals, which are a prominent factor of individual differences (Friedman et al., 2008; Miyake and Friedman, 2012; Friedman and Miyake, 2017). Accordingly, some of the individuals might be more able than others to flexibly adjust their cognitive control mechanisms such as increasing or decreasing inhibition of distractors or to maintain more or less information concurrently to process multiple tasks. Second, the level of response organization might be rather affected by previous experiences with situations demanding for multitasking, knowledge gained from the media or science about multitasking, or attitudes perceived in a persons’ cultural environment (see e.g., González and Mark, 2004; Horrey et al., 2008; Ophir et al., 2009; Poposki and Oswald, 2010; Dabbish et al., 2011; Lohmann-Haislah, 2012). Complementary, the different degrees of stability might be related to the fact that the task coordination levels are accessible to different degrees. Obviously, the behavioral level of task coordination, as it reflects voluntary actions, is more accessible to conscious reflection than the task processing level. In this sense, it might be more strongly affected by the individual’s attitude toward multitasking than the individual’s cognitive processing style and rather constitutes a form of habit, inherently characterized by a high stability.

## Data Availability Statement

The raw data supporting the conclusions of this article will be made available by the authors, without undue reservation.

## Ethics Statement

The studies involving human participants were reviewed and approved by the Ethics Committee of the Department of Psychology and Ergonomics at the Technische Universität Berlin. The patients/participants provided their written informed consent to participate in this study.

## Author Contributions

RR and JB contributed to conception and design of the study, performed the statistical analysis, and wrote sections of the manuscript. RR collected the data and wrote the first draft of the manuscript. Both authors contributed to manuscript revision, read, and approved the submitted version.

## Conflict of Interest

The authors declare that the research was conducted in the absence of any commercial or financial relationships that could be construed as a potential conflict of interest.

## Publisher’s Note

All claims expressed in this article are solely those of the authors and do not necessarily represent those of their affiliated organizations, or those of the publisher, the editors and the reviewers. Any product that may be evaluated in this article, or claim that may be made by its manufacturer, is not guaranteed or endorsed by the publisher.

## Footnotes

1
https://osf.io/e6wgx/

2
https://osf.io/sb6wq/

3 According to classification criteria, this participant showed a clear pattern of response grouping in the condition of high risk of crosstalk. However, in the condition of low risk of crosstalk this participant seemed to switch most of the time with interspersed brief phases of response grouping.

4 German pronunciation: D: [de:], P: [pe:], T: [te:], W: [ve:] vs. H: [ha:], K: [ka:], J: [jt], Q: [ku:].

5 Note that, since participants are free to respond to either of the two tasks at any time during the FCDT, an RT measure describing the time between stimulus onset and response input does not accurately reflect response timings in this context: While the stimulus for task A is visible, participants could choose to respond to task B once or multiple times before attending to task A again.

6 This criterion was adopted in line with the suggestions by Berger and Kiefer (2021). Note, however, that a criterion of 2.5 or 3 SDs, respectively, yielded no differences in the result patterns.

7 A reviewer suggested to test the effect of risk of crosstalk on the overall sample. That is, including the individuals, who shifted in their preferred response strategy. Indeed, the according paired *t*-test yielded a significant result: *t*(52) = −2.22, *p* = 0.031, $\hat{d}$ = −0.30. However, a closer inspection of the differences in switch rates due to crosstalk showed that this effect is driven by just three of the 53 individuals (see Supplementary Figure 2). They exhibited extreme values in their switch rate differences (−35.5%, −39%, and −72.7%) as opposed to the remaining sample (*M* = −1.4%, *SD* = 6.5%). An according *t*-test omitting these three participants was again non-significant: *t(*49) = −1.50, *p* = 0.139, $\hat{d}$ = −0.21. The corresponding Bayes factor provides anecdotal evidence for the null, *BF*_01_=2.27.

8 Tests for differences in switch rates between groups were not conducted, as those differences in switch rates are largely predetermined by the way in which the strategies are identified in the first place. Thus, no viable insights were expected from comparing mean switch rates between strategies in the context of this study.

9 Note that, exploratory analyses of linear relationships between MPI score and mean switch rates within the groups of interleaver, or switcher alone, were also non-significant in both crosstalk conditions.

10 Noteworthy, in the present study the group of individuals favoring response grouping was comparably small. This might be due to random sampling, as the preferences’ natural occurrence is assessed (i.e., individual differences cannot be assigned to participants) or due to slight differences in the studies’ design. However, as to why exactly the group of response grouper was so small remains a matter of speculation.

## Appendix

### Performance Efficiency

The overall dual-tasking performance efficiency (in short: ODTPE) is a throughput measure that facilitates the assessment of multitasking costs or benefits by comparing performances in single- and dual-task conditions. It was first introduced by Reissland and Manzey (2016) and refined by Brüning et al. (2021). The metric is formally defined as follows:

$$O\,D\,T\,P\,E=100\times \left[\left(\frac{\left(n\,C_{A\,\_\,d\,u\,a\,l}\right)}{\left(n\,C_{A\,\_\,s\,i\,n\,g\,l\,e}\right)}+\frac{\left(n\,C_{B\,\_\,d\,u\,a\,l}\right)}{\left(n\,C_{B\,\_\,s\,i\,n\,g\,l\,e}\right)}\right)/2\right]-100$$

The variables nC_A_single_ and nC_B_single_ represent the number of correct responses for tasks A and B in single-task conditions. The number of correct responses for each task in dual-task conditions is given by nC_A_dual_ and nC_B_dual_, respectively. Thus, the recorded performance for dual-tasking is compared to the number of correct responses that would theoretically be expected if participants were to process the tasks strictly separately. Conveniently, the ODTPE score reflects the percentage deviation in dual-task from single-task performance which serves as reference. A score > 0 indicates performance benefits in multitasking, while performance losses are reflected by values <0. Note that, this metric requires that the time participants spend performing the dual-task blocks must be the sum of the durations of the single-task blocks.

As an illustration, consider the following example: In the single-task condition, a participant performs tasks A and B separately for 1 min each. The number of correct responses is nC_A_single_ = 60 for task A and nC_B_single_ = 70 for task B. In a 2-min dual-task block, during which the participant has to switch between the two tasks at regular intervals, he scores 55 right answers for task A and 60 for task B (nC_A_dual_ = 55, nC_B_dual_ = 60). In this case, ODTPE equals –11.3%, which indicates a performance loss in the multitasking-situation.
