# Supplementary material for: Individual Strategies of Response Organization in Multitasking Are Stable Even at Risk of High Between-Task Interference
Source: Front Psychol. 2022 Apr 6;13:860219. doi: 10.3389/fpsyg.2022.860219 (PMC9019473; doi:10.3389/fpsyg.2022.860219)
Supplement: Supplementary file 1 [file Table_1.DOCX]

**Figure S1**

*Relationship Between Individual Switch Rates in Percentage and the Multitasking Preference Inventory (MPI) Score for Both Crosstalk Conditions*


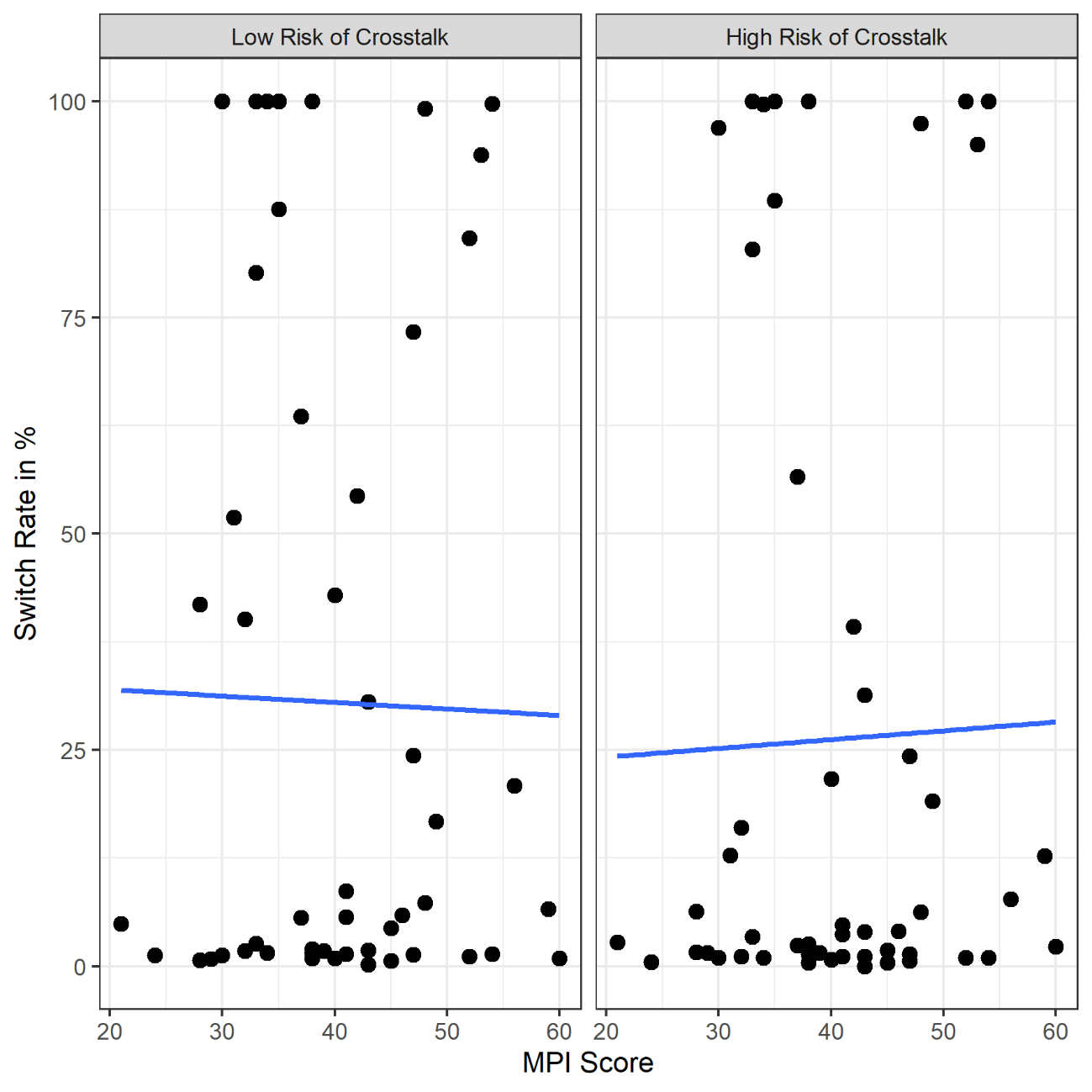


*Note.* Switch rates indicate the number of switches performed relative to the total number of possible switches. The MPI score reflects the degree of polychronicity, with higher scores indicating that individuals are more likely to be polychronic.

**Figure S2**

*Differences in Individual Switch Rates Between Crosstalk Conditions in Percentage*


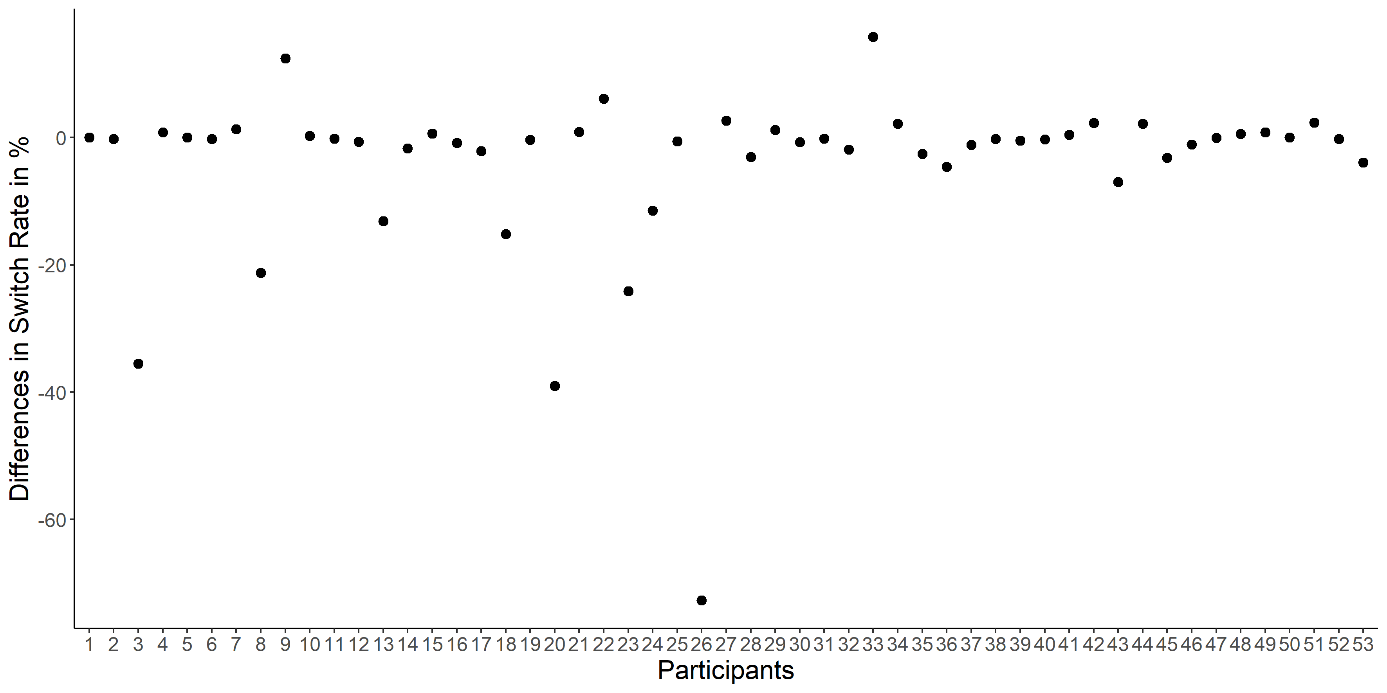


*Note.* Switch rates indicate the number of switches performed relative to the total number of possible switches. Switch rates observed under the low risk of crosstalk condition were subtracted from those obtained under the high risk of crosstalk condition (i.e., negative values indicate a decrease in switch rates from the low to the high risk of crosstalk condition).
